# Choosing Appropriate Nutritional Therapy for Patients With Anorexia Nervosa Exhibiting Liver Dysfunction: A Case Report

**DOI:** 10.7759/cureus.54332

**Published:** 2024-02-16

**Authors:** Motoharu Tsutsumi, Naomichi Okamoto, Hirofumi Tesen, Reoto Kijima, Reiji Yoshimura

**Affiliations:** 1 Psychiatry, University of Occupational and Environmental Health, Kitakyushu, JPN

**Keywords:** nutritional therapy, liver dysfunction, refeeding syndrome, autophagy, anorexia nervosa

## Abstract

Anorexia nervosa (AN) presents with a variety of physical complications such as hypoglycemia, electrolyte abnormalities, and dehydration associated with starvation, requiring rapid weight gain through nutritional therapy. However, despite nutritional therapy, patients are at risk of many serious medical complications, including hypoglycemia, hypophosphatemia, edema, and liver damage. Starvation has been found to cause hepatocyte injury with mild-to-severe increases in liver enzyme levels, and distinguishing between autophagy and refeeding syndrome is important for treatment strategies. Herein, we report a rare case of sudden liver injury after the initiation of nutritional therapy in a patient with AN. A 35-year-old woman was admitted to our hospital for the treatment of weight loss due to AN. Nutritional therapy was initiated at 600 kcal/day and increased to 1500 kcal/day on the 21st day of admission. On the 22nd day after admission, rapid liver injury was observed, with an aspartate aminotransferase level of 141 U/L and an alanine aminotransferase level of 221 U/L. After the exclusion of refeeding syndrome, since there was no evidence of hypokalemia, hypophosphatemia, or fatty liver disease based on blood tests and abdominal echography, we diagnosed starvation-induced hepatocyte autophagy, and she was treated with the same calories. Her liver dysfunction gradually improved thereafter. This case report highlights the clinical utility of identifying the etiology of hepatic dysfunction in patients with AN. Clinicians must make appropriate decisions regarding continuing or reducing nutritional therapy based on relevant tests when patients with AN develop liver dysfunction after the initiation of nutritional therapy.

## Introduction

Anorexia nervosa (AN) is a life-threatening disease with high morbidity and mortality rates. AN is characterized by restricted energy intake, weight loss, fear of weight gain, and a distorted body image [1]. AN presents with various physical complications, such as hypoglycemia, electrolyte abnormalities, and dehydration, due to extreme undernutrition [2].

Starvation has been found to cause hepatocyte injury with mild-to-severe increases in liver enzyme levels [3,4]. Although the exact pathophysiology of hepatic dysfunction associated with AN is unknown, patients with AN are known to have hepatic dysfunction with autophagy [5]. Nutritional therapy can also cause hepatic dysfunction due to refeeding syndrome; therefore, clinicians need to perform proper physical evaluations and identify the cause of abnormal findings.

Reporting appropriate nutritional therapy is vital as being underweight associated with AN may have potentially lethal consequences. During nutritional therapy for AN, it is vital to differentiate between refeeding syndrome and autophagy; however, few case reports have addressed the actual differentiation and treatment processes involved. Here, we report the case of a patient with AN who developed hepatic dysfunction after the initiation of nutritional therapy, which gradually improved with continued nutritional therapy.

## Case presentation

A 35-year-old woman was admitted to our hospital for the treatment of weight loss associated with AN. The patient had no family history of psychiatric illnesses and did not take any medications.

At the age of 22 years, the patient began to fear being overweight and to self-induce vomiting, resulting in weight loss from 61 kg to 48 kg (height: 160.9 cm, body mass index (BMI): 18.5 kg/m^2^). At the age of 35 years, her caloric intake at home was approximately 400 kcal/day. Her weight decreased to 30.6 kg (BMI: 11.8 kg/m^2^), and she was admitted to our hospital.

The patient was unaware of her illness and showed fear of weight gain and a distorted body image. On admission, blood tests revealed mildly elevated levels of liver enzymes, with aspartate aminotransferase (AST), 35 U/L; alanine aminotransferase (ALT), 45 U/L; and hypokalemia, 2.2 mmol/L. Her thiamine level was normal (25.7 ng/mL). Blood tests were negative for hepatitis B surface antigen, hepatitis C virus antibody, antinuclear antibody, and anti-mitochondrial antibody. Electrocardiography revealed bradycardia with a heart rate of 54 bpm; however, no other abnormalities were noted. She was diagnosed with AN based on the diagnostic and statistical manual of mental disorders-5. Nutritional therapy was initiated at a dose of 600 kcal/day (constituents: 65% carbohydrate, 15% protein, 20% fat) three times a day (morning, noon, and evening) orally. Potassium gluconate 24 mEq/day was initiated, and the potassium levels normalized by day 8. From the 10th day of hospitalization, nutrient intake reached 1000 kcal/day (14th day of hospitalization) and was increased to 1500 kcal/day (21st day of hospitalization).

**Figure 1:**
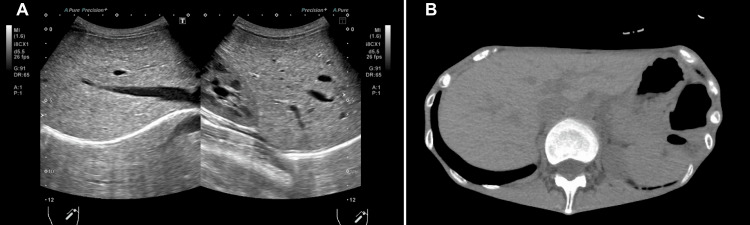
Abdominal ultrasonography and CT (A) Abdominal ultrasonography. (B) CT demonstrates no hepatic tumors, hepatomegaly, or fatty liver. CT, computed tomography.

On day 22 of hospitalization, blood tests revealed rapid liver damage, with an AST level of 221 U/L and an ALT level of 141 U/L. Blood test results were negative for autoimmune and viral hepatitis. She had edema and constipation, but no vomiting or nausea. Abdominal ultrasonography and CT tomography revealed no liver tumors, hepatomegaly, or fatty liver disease (Figure 1). 

Although refeeding syndrome was considered, liver dysfunction after the initiation of nutritional therapy was excluded because there was no exacerbation of hypokalemia or hypophosphatemia, and no fatty liver was observed. Starvation-induced hepatocyte autophagy was suspected, and nutritional therapy (1500 kcal/day) was continued. On day 43 of hospitalization, the caloric intake was increased to 2500 kcal/day, weight improved to 37.2 kg (BMI: 14.4 kg/m^2^), AST and ALT levels were 26 U/L and 86 U/L, respectively, and the liver injury gradually improved (Table 1). Subsequently, the patient was transferred to another hospital for further management.

**Table 1:** Blood data summary ALT, alanine aminotransferase; AST, aspartate aminotransferase; BMI, body mass index.

	Hospitalized	Appearance of liver dysfunction	Discharge from hospital	Reference range
Body weight (kg)	30.6	35.1	37.3	-
BMI (kg/m^2^)	11.8	13.6	14.4	18.5 to 25
AST (U/L)	35	141	26	10 to 40
ALT (U/L)	45	221	86	5 to 40
Sodium (mEq/L)	138	141	142	136 to 147
Potassium (mEq/L)	2.2	3.9	4.1	3.6 to 5.0
Phosphorus (mEq/L)	3.9	3.8	3.7	2.4 to 4.3
Thiamine (ng/mL)	25.7	-	-	24 to 66

## Discussion

We report the case of a patient with AN who developed hepatic dysfunction after the initiation of nutritional therapy, which gradually improved with continued nutritional therapy. This case report highlights the clinical utility of identifying the etiology of hepatic dysfunction treatment in patients with AN.

AN adversely affects various organs and tissues, such as the liver, heart, bones, and blood, due to weight loss and undernutrition. Furthermore, it can cause diverse physical complications such as liver dysfunction, bradycardia, osteoporosis, blood cell loss, hypoglycemia, and electrolyte abnormalities [2]. Therefore, rapid weight gain through nutritional therapy is necessary; however, the risk of many serious medical complications, such as hypoglycemia, hypophosphatemia, edema, and liver damage, is recognized even after nutritional therapy is initiated [6]. 

The pathophysiology of the liver dysfunction associated with AN is unknown; however, mechanisms such as hepatic autophagy, acute hypoperfusion, and refeeding syndrome have been proposed. Autophagy is an intracellular process that degrades excess or abnormally long-lived cytoplasmic proteins and organelles within lysosomes, removes the resulting macromolecules, and ultimately recycles them [7]. During starvation, autophagy initially acts in a hepatoprotective manner by preventing hepatocyte necrosis and liver failure. However, when starvation persists, excessive autophagy is no longer protective and degrades liver proteins in response to nutrient depletion, leading to hepatocellular death and liver failure [5]. In acute hypoperfusion, hypotension due to hypoglycemia and relative bradycardia causes decreased blood flow to the liver and increased cell-death-derived liver enzymes [8]. In refeeding syndrome, the rapid influx of glucose into the bloodstream increases insulin secretion, leading to fatty liver disease and elevated liver enzyme levels due to the accumulation of fat in hepatocytes [9]. 

During nutritional therapy for AN, it is vital to differentiate between refeeding syndrome and autophagy, as this is relevant to the treatment strategy. Autophagy in the liver is suppressed mainly by amino acids via activation of mTORC1 [10]. Improvement of liver function with appropriate nutritional therapy in AN supports autophagy as the primary cause of elevated hepatic enzymes [5]. However, there have been reports of further exacerbation of autophagy-induced liver dysfunction even after the initiation of nutritional therapy, as in this case [11]. The reason for this is unknown. However, in this case, the total caloric intake was possibly insufficient immediately after the start of nutritional therapy, or liver function took a long time to improve and liver damage may have occurred after the start of nutritional therapy. 

Autophagosomes are detected via liver biopsy, whereas hepatomegaly and fatty liver are observed via abdominal echography in patients with refeeding syndrome. However, liver biopsy is not actively recommended because it is invasive and carries the risk of complications such as pain, bleeding, and puncture of other organs [12]. Therefore, differentiation based on blood tests and imaging findings is crucial. In this case, since there was no evidence of hypokalemia, hypophosphatemia, or fatty liver disease on blood tests and abdominal echography, liver damage was considered to be due to autophagy rather than refeeding syndrome. Therefore, we continued nutritional therapy at the same calorie level. We have shown the types and differentiation of liver dysfunction in Table 2. 

**Table 2:** Types and differentiation of liver dysfunction

Liver dysfunction	Method of identification
Drug-induced liver injury	Check internal medications
Viral hepatitis	Determination of viral antigens and corresponding antibodies
Autoimmune hepatitis	Determination of antibodies
Alcoholic hepatitis	Check alcohol consumption
Starvation-induced hepatocyte autophagy	Liver biopsy shows autophagosomes
Refeeding syndrome	Blood tests show hypokalemia and hypophosphatemia, and abdominal imaging shows fatty liver disease

Liver damage due to refeeding syndrome requires slowing down of nutritional therapy [9], whereas autophagy improves with continued nutritional therapy [13]. Thus, clinicians need to select conflicting treatments based on appropriate tests when patients with AN present with liver dysfunction during nutritional therapy. This case suggests its clinical utility in treatment.

## Conclusions

Herein, we report a case of rapid liver dysfunction following the initiation of nutritional therapy in a patient with AN. This case report highlights the clinical utility of identifying the etiology of hepatic dysfunction in patients with AN. During nutritional therapy for AN, it is vital to differentiate between refeeding syndrome and autophagy, as this is relevant to the treatment strategy. Refeeding syndrome was ruled out based on blood tests, imaging studies, and continued nutritional therapy, and liver biopsy was not essential in this case. Clinicians must make appropriate choices that conflict with continuing or reducing nutritional therapy based on appropriate tests when patients with AN present with liver dysfunction after initiation of nutritional therapy.
